# Natural outcome of hemoglobin and functional recovery after the direct anterior versus the posterolateral approach for total hip arthroplasty: a randomized study

**DOI:** 10.1186/s13018-020-01716-4

**Published:** 2020-06-01

**Authors:** Jia Cao, Yiqin Zhou, Wei Xin, Jun Zhu, Yi Chen, Bo Wang, Qirong Qian

**Affiliations:** Department of Joint Surgery and Sports Medicine, Shanghai Changzheng Hospital, Second Military Medical University, No.415, Fengyang Road, Shanghai, 200003 China

**Keywords:** Total hip arthroplasty, Direct anterior approach, Posterolateral approach, Hemoglobin, Functional recovery

## Abstract

**Background:**

Total hip arthroplasty (THA) is one of the most successful orthopedic surgeries. There are many common surgical approaches for THA. The direct anterior approach (DAA) and posterolateral approach (PLA) were compared, leading to controversial results.

**Methods:**

We report on a prospective randomized study which compared the changes of perioperative hemoglobin (Hb), the Harris hip score (HHS) and a visual analog scale (VAS) pain score following THA using DAA or PLA. A total of 130 participants were randomly divided into two groups (65 DAA versus 65 PLA). Perioperative ΔHb and other clinical outcomes were recorded.

**Results:**

A total of 130 participants completed follow-up, while 14 patients were not recorded in blood outcomes due to blood transfusions and complications. The average Hb decrease immediately after surgery in the DAA group was greater than that in the PLA group (21.1 versus 15.8 g/L, *P* < .001). However, post-operative Hb descent velocity was slower in the DAA group, and the lowest point was reached earlier. No significant differences in ΔHb levels could be observed after 1 month in the two groups. When compared with the PLA group, the DAA group had a shorter incision (9.1 versus 13.5 cm, *P* < .001) and shorter hospital stay (4.2 versus 4.7 days, *P* = .004). However, the operation time of the DAA group was longer (88.0 versus 66.8 min, *P* < .001). The DAA group had a better HHS and VAS pain score at 6 weeks post-surgery. However, no significant differences were observed at later time points.

**Conclusion:**

We concluded that DAA performed better on enhanced recovery after surgery (ERAS) than PLA in THA, while both DAA and PLA could result in a positive, similar result after 3 months.

**Trial registration:**

The study was registered by the Chinese Clinical Trial Registry (ChiCTR1900020770, 19 January 2019).

## Background

Total hip arthroplasty (THA) has been proven to be a successful treatment for hip conditions such as developmental dysplasia, osteonecrosis of the femoral head, and hip osteoarthritis, and can result in relief pain, improved function and enhanced quality of life [1, 2]. Over 400,000 total hip arthroplasties are performed in China each year. And significant increases in use of THA are expected in China in the future, if the current trend continues [3]. However, some patients still experience pain associated with surgical trauma after THA. In order to reduce this trauma, surgeons are exploring minimally invasive approaches on the basis of traditional surgical approaches of THA, including the anterior approach, the lateral approach, the posterolateral approach (PLA), the Hardinge approach and the Watson-Jones approach. Additionally, a new series of minimally invasive surgical approaches has been proposed, including the posterior two incision approach, the direct anterior approach (DAA) and the supercapsular percutaneously assisted total hip approach [4–6]. DAA is being used for more patients due to its shorter length of incision, reduced soft tissue damage and faster recovery. Further, many studies have reported that DAA is associated with better outcomes of gait analysis, muscle damage, early functional recovery, and pain relief [7–10]. Conversely, some researchers have pointed out increased complication rates of DAA during the learning curve [11–13]. A study has reported that DAA was associated with higher complication rate in the early phase [14]. And another study has pointed out the risk of revision was higher with DAA, especially due to stem loosening [15].

With the number of THA that are performed in China increasing dramatically, it is more and more important to develop feasible strategies to improve medical quality of patients undergoing THA to obtain better clinical outcomes. Enhanced recovery after surgery (ERAS) is proposed as a series of evidence-based perioperative optimizations with multidisciplinary approach to reduce surgical stress and accelerate postoperative recovery [16]. ERAS pathway for THA has been reported to promote earlier recovery and be beneficial for patients [17]. Trauma that has been caused by THA can consume a large amount of hemoglobin (Hb), with postoperative Hb decline potentially reaching 30–40 g/L [18]. Postoperative Hb decline leads to an increase in the perioperative blood transfusion rate, which in turn leads to greater costs, prolonged hospital stays, increased complications, and transfusion reactions [19, 20]. At present, articles on perioperative blood management in THA tend to focus on the use of tranexamic acid as well as whether or not to use drainage tubes [21, 22]. While some studies have compared amounts of blood loss in different approaches, there have been few reports of changes in the perioperative Hb. An understanding of the changes in Hb during the perioperative period of THA could help control blood transfusion indications as well as further achieving fast track arthroplasty. Here, we observed Hb changes in the perioperative period of the two approaches (DAA and PLA), and then compared the early recovery period of the approaches.

## Methods

This single-center prospective cohort study took place between March and June 2019. Patients undergoing primary total hip arthroplasty at Shanghai Changzheng Hospital were asked to participate. The study was approved by our institutional review board and registered by the Chinese Clinical Trial Registry (ChiCTR1900020770). Written informed consent was obtained from all participants. Inclusion criteria were as follows. Patients had to be diagnosed as having osteoarthritis, femoral head necrosis or developmental dysplasia of the hip (Crowe I–II). Patients had to have a body mass index (BMI) of no more than 30 kg/m^2^ with no obvious osteoporosis and the American Society of Anesthesiologists score no more than 3. Patients must not have anemia (male Hb > 120 g/L, female Hb > 110 g/L), blood system diseases or systemic inflammatory (rheumatic) diseases. They also must not have any previous history of hip surgery or infection. If patients had received intraoperative or postoperative blood transfusions, their blood outcome results would not be recorded, while relevant blood transfusion records would be written. Patients with severe postoperative complications, such as prosthetic loosening and revision, periprosthetic fractures, postoperative infections, nerve and vascular injuries, perioperative deep vein thrombosis, pulmonary embolism, or cardiovascular accidents would also not be recorded in blood outcomes. Any complications experienced by participants would be recorded. Patients who met the inclusion criteria were assigned to the DAA or PLA group by choosing closed envelopes which contained random numbers. Both preoperative and postoperative data were collected by an independent researcher. Due to obvious difference in surgical incisions, we did not use blind methods. All operations were completed by three experienced surgeons, each of whom had completed more than 100 DAA THA and 100 PLA THA using cementless prostheses (that is, LCU, Link, or Corail, DePuy). All the patients used the same type of cementless protheses (Pinnacle + Corail, DePuy Synthes, USA) in this study. And preoperative template measurement was performed before each operation.

### Direct anterior approach: surgical technique

In this approach, the patient is positioned in a supine position on a regular operating table. A skin incision, around 8 cm long, is made along the inferolateral of the anterior superior iliac spine, towards the fibular head. The anterior hip capsule is exposed through the space between the tensor fascia lata and the rectus femoris. The ascending branch of the lateral femoral artery is found and ligation performed while it is exposed. After opening the hip capsule anteriorly, a measured femoral neck osteotomy is performed, based on results of preoperative template measurement, after which the femoral head is removed. After this, the acetabular reaming is performed and the acetabular component inserted. The operative limb is sufficiently externally rotated, adducted and stretched. The femoral canal is broached to the appropriate size, using the hook to raise the proximal femur for optimal exposure and operation. The femoral implant and head are placed following a trial reduction using the femoral implant trial to ensure leg length and offset suitability. During surgery, fluoroscopy is utilized to verify the position of the acetabular and femoral components as well as leg length, and then to offset. Finally, the articular capsule and incision are stitched.

### Posterolateral approach: surgical technique

In this technique, the patient is positioned in the lateral decubitus position on a regular operating table. A 10–15 cm curvilinear incision is placed over the greater trochanter at the posterolateral aspect of the hip. A blunt dissection of gluteus maximus in line with its fibers is executed in order to reach the short external rotators and open the posterior capsule. A femoral neck osteotomy is then performed following the posterior dislocation of the hip joint. The acetabular and femur are prepared, and these components are then inserted into the appropriate location after trialing. The C-arm is used to confirm leg length and offset. Finally, the articular capsule is repaired, but the external rotator is not reconstructed in the muscle group. Closure is performed as standard.

### Perioperative management

Postoperative patients were treated with analgesia, anticoagulation, infection prevention (using antibiotics once following surgery), rehydration, and symptomatic support. None of patients were given a drainage tube, while rivaroxaban was used for anticoagulation 1 month after surgery. Blood transfusions indicated that after hip replacement, this center is Hb < 80 g/L [23]. However, some patients and their families felt concerned about the risk of blood transfusion. Blood transfusion indications for patients with no obvious anemia symptoms after surgery were adjusted to Hb < 75 g/L. These patients were then closely observed. All patients were encouraged to get out of bed on the day of surgery and start weight-bearing walking with the help of walking aids in the following days. Both groups had the same postoperative functional rehabilitation protocols. Patients of the PLA group were asked to avoid flexing their hip joints to more than 90° or adducting their hip joints beyond neutral. Patients in the DAA group had no range of motion restrictions. Patients with no serious complications or obvious anemia were discharged from hospital. In addition, patients were told that they could stop using the walking aids gradually after being discharged from the hospital and that activities which did not lead to discomfort were preferred.

### Outcome measures

Participants were evaluated preoperatively, immediately after the surgery and at 1 to 5 days, 1 week, 2 weeks, 3 weeks, 6 weeks, 3 months, and 6 months postoperatively following THA. Primary outcome measures were the Hb immediately after surgery and at 1 to 5 days, 2 weeks, 6 weeks, 3 months, and 6 months postoperatively. Secondary outcome measures included the Harris hip score (HHS) and a visual analog scale (VAS) pain score at 1 week, 3 weeks, 6 weeks, 3 months, and 6 months postoperatively. Surgical data such as operative time, wound length and length of hospital stay was also recorded. Data from all participants were collected by the same investigator.

### Statistical analysis

Statistical analysis was performed with SPSS 20.0. Results were reported as mean ± standard deviation (SD) as appropriate. Participants’ demographic characteristics were compared with a Pearson chi-squared test. Group *t* tests were used for comparing clinical data when normality (and homogeneity of variance) assumptions were satisfied. For groups in which this was not the case, the equivalent non-parametric test was used. Levene’s test was used to measure homogeneity of variance. All statistical tests were two-sided, and the statistical significance level was 5%, with *P* values less than 0.05 considered statistically significant.

## Results

**Fig. 1:**
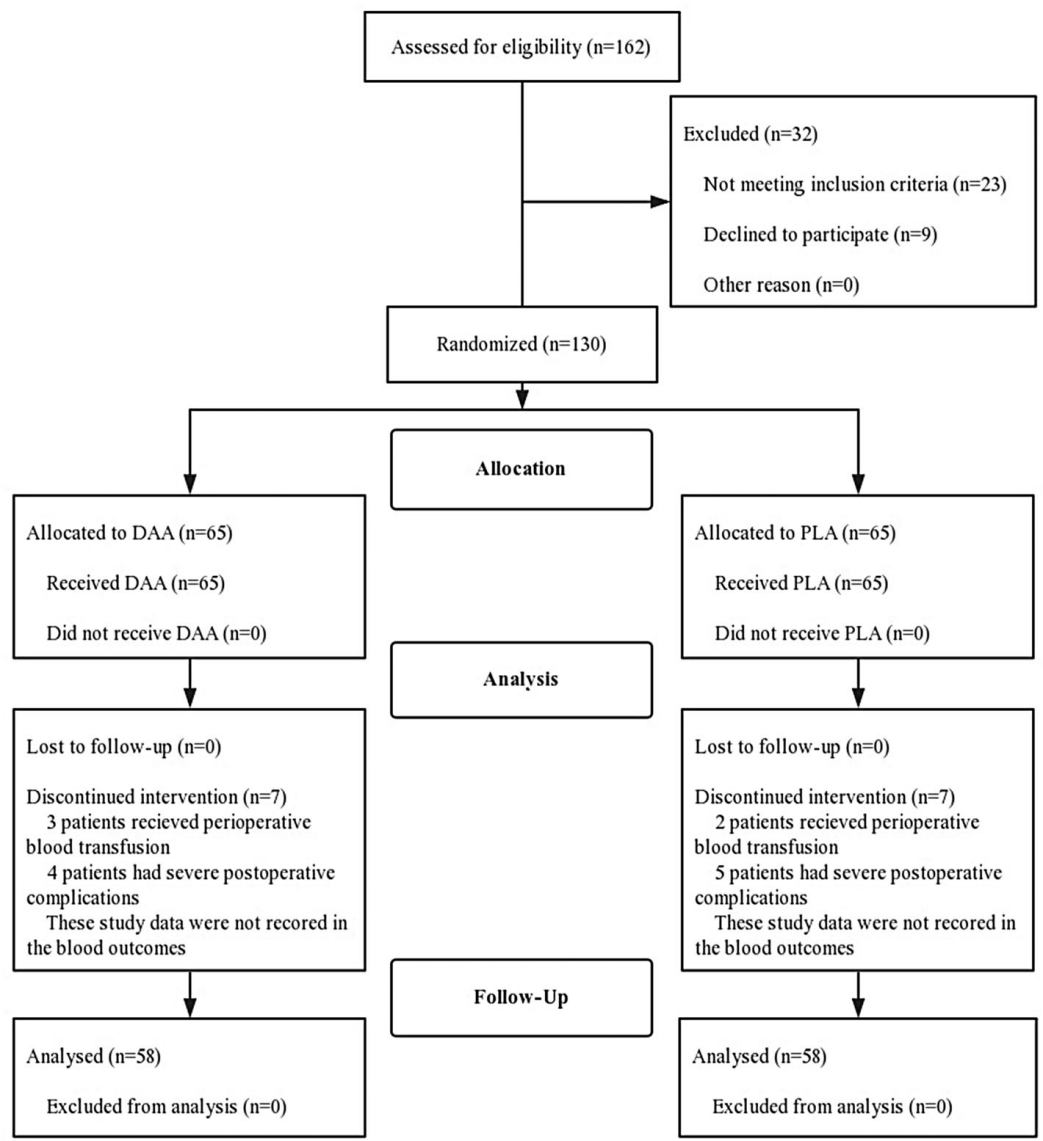
Consort diagram of patient enrollment characteristics. DAA, direct anterior approach; PLA, posterolateral approach

One hundred and thirty patients were recruited and randomly divided into two groups. Sixty-five patients were assigned to the DAA group, while the others were assigned to the PLA group. A total of 130 patients were followed up. Fourteen patients were not involved in the statistical analysis of blood outcomes due to postoperative blood transfusions and complications; these people totaled seven in the DAA group and seven in the PLA group (see Fig. 1, Table 4). In the DAA group, there were 27 men and 38 women, who had a mean age of 61.4 years and a mean BMI of 24.7 kg/m^2^. In the PLA group, there were 28 men and 37 women, who had a mean age of 62.4 years and a mean BMI of 25.1 kg/m^2^. No statistically significant differences in the demographic characteristics of the two groups were found (see Table 1). Both the DAA and PLA groups appeared to be well matched for preoperative data, and no measurable differences were found in the preoperative data (see Table 1).

**Table 1:** Demographic characteristics and preoperative data

Variable (mean ± SD)	DAA	PLA	*P* value
Age, years	61.4 ± 12.8	62.4 ± 8.3	0.564
Males/females	27/38	28/37	0.859
BMI, kg/m^2^	24.7 ± 1.9	25.1 ± 1.8	0.180
Hb, g/L	131.7 ± 9.9	133.4 ± 6.3	0.228
HHS	45.8 ± 4.0	46.8 ± 6.5	0.272
VAS	5.9 ± 1.3	6.2 ± 1.1	0.085

### Primary outcomes

**Fig. 2:**
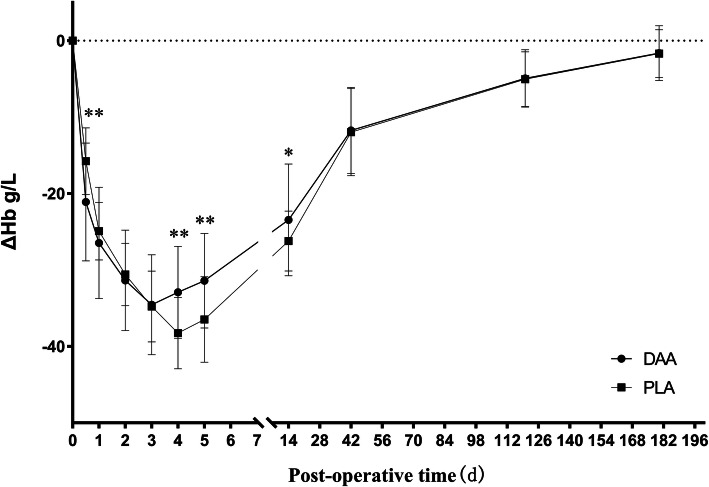
Postoperative level of ΔHb, *P < 0.05, **P < 0.01

We used ΔHb, which measured the difference between Hb at a certain time point and preoperative Hb, to indicate changes of perioperative Hb. The preoperative Hb of the DAA group was 132.1 ± 10.2 g/L. In this group, Hb decreased significantly from the day of surgery to the third day after surgery. The decline was most obvious on the day following surgery, with an absolute value of ΔHb of 21.1 ± 7.7 g/L (see Table 2, Fig. 2). The lowest Hb value was reached on the third day following surgery, while Hb began to rise on the fourth day following surgery for the DAA group (see Table 2, Fig. 2). In this same group, at 2 weeks postoperatively, Hb increased from the lowest point to 82.3% of the preoperative level (see Table 2, Fig. 2). Hb then returned to a preoperative level 3 months after surgery and remained at a preoperative level until 6 months after surgery for the DAA group (see Table 2, Fig. 2).

**Table 2:** ΔHb at different time points

Variable (mean ± SD)	DAA	PLA	*P* value
ΔHb^*^, g/L			
Post-op, day of surgery	− 21.1 ± 7.7	− 15.8 ± 4.4	< .001
1 day post-op	− 26.5 ± 7.3	− 24.9 ± 3.8	0.154
2 days post-op	− 31.4 ± 6.6	− 30.6 ± 4.1	0.448
3 days post-op	− 34.6 ± 6.5	− 34.8 ± 4.6	0.832
4 days post-op	− 32.9 ± 6.0	− 38.3 ± 4.7	< .001
5 days post-op	− 31.4 ± 6.2	− 36.5 ± 5.6	< .001
2 weeks post-op	− 23.4 ± 7.3	− 26.2 ± 3.9	0.013
6 weeks post-op	− 11.7 ± 5.6	− 12.0 ± 5.7	0.832
3 months post-op	− 4.9 ± 3.8	− 5.0 ± 3.6	0.900
6 months post-op	− 1.6 ± 3.6	− 1.7 ± 3.1	0.934

^*^The difference between Hb at a certain time point and preoperative Hb

In contrast, the preoperative Hb of the PLA group was 134.2 ± 5.7 g/L. In this group, Hb decreased significantly from the day of surgery to the fourth day following surgery. This decline was most obvious on the day following surgery, with an absolute value of ΔHb of 15.8 ± 4.4 g/L (see Table 2, Fig. 2). The lowest point of Hb was reached on the fourth day after surgery, while Hb began to rise on the fifth day after surgery for this group (see Table 2, Fig. 2). At 2 weeks following surgery, Hb for this group increased from the lowest point to 80.5% of preoperative level (see Table 2, Fig. 2). Hb then returned to the preoperative level 3 months following surgery and remained at preoperative level until 6 months after surgery (Table 2, Fig. 2). Four patients had a postoperative Hb minimum of between 75 g/L and 80 g/L, with no blood transfusion performed after surgery. All of these patients recovered to more than 85% of their preoperative level 6 weeks after surgery.

Statistically significant primary outcomes which were associated with the DAA group included a further drop in Hb on the day of surgery (*P* < .001), and a reduced drop in Hb on the fourth and fifth days after surgery (*P* < .001) as well as the 2 weeks after surgery (*P* = 0.013) when compared of the PLA group. No statistically significant differences were found in ΔHb between both groups at other time points (Table 2).

### Secondary outcomes

Statistically significant differences such as operative time, wound length and length of hospital stay were found between the two groups (see Table 3). The operative time for the DAA group was found to be 88.0 ± 4.5 min compared to 66.8 ± 4.5 min for the PLA group (*P* < .001). Patients from the DAA group had a shorter incision than those in the PLA group (*P* < .001). The DAA group was discharged sooner than patients in the PLA group (*P* = 0.004). Patients of both groups had similar HHS and VAS pain scores before the surgery. However, patients from the DAA group recovered faster and had less pain than those in the PLA group. The HHS for the DAA group was 78.7 ± 3.3 compared to 71.7 ± 4.1 for the PLA group (*P* < .001), while the VAS pain score for the DAA group was found to be 2.1 ± 0.7 compared to 3.0 ± 0.7 for the PLA group (*P* < .001) at 1 week post-surgery (Table 3). Similarly, the DAA group had a higher HHS and a lower VAS pain score at three (*P* < .001, *P* < .001) and 6 weeks (*P* < .001, *P* < .001) post-surgery (Table 3). No statistically significant differences in HHS and VAS pain score were found at 3 or 6 months postoperatively (Table 3).

**Table 3:** Surgical and postoperative data

Variable (mean ± SD)	DAA	PLA	P-Value
Operative time, min	88.0 ± 4.5	66.8 ± 4.5	< .001
Wound length, cm	9.1 ± 0.6	13.5 ± 0.9	< .001
Length of hospital stay, day	4.2 ± 1.0	4.7 ± 0.7	0.004
HHS			
1 week post-op	78.7 ± 3.3	71.7 ± 4.1	< .001
3 weeks post-op	84.2 ± 3.4	77.2 ± 3.2	< .001
6 weeks post-op	88.7 ± 2.5	80.0 ± 2.6	< .001
3 months post-op	91.6 ± 1.1	91.3 ± 1.3	0.100
6 months post-op	93.0 ± 1.5	92.9 ± 1.4	0.672
VAS			
1 week post-op	2.1 ± 0.7	3.0 ± 0.7	< .001
3 weeks post-op	1.0 ± 0.6	1.7 ± 0.8	< .001
6 weeks post-op	0.5 ± 0.5	0.9 ± 0.8	< .001
3 months post-op	0.3 ± 0.5	0.4 ± 0.5	0.599
6 months post-op	0.2 ± 0.4	0.2 ± 0.4	0.680

Five patients underwent postoperative blood transfusion: three in the DAA group and two in the PLA group (see Table 4). Nine patients experienced postoperative complications (Table 4), while postoperative dislocation occurred in two patients from the PLA group. Both of them were treated with closed reduction and had no further sequelae. One patient in the PLA group suffered periprosthetic fractures due to an accidental fall after discharge and had a revision operation. One episode of trochanteric bursitis was resolved by a cortisone injection for a patient in the PLA group. Complications in the DAA group included three cases of lateral femoral cutaneous nerve injury. Their symptoms gradually improved and did not receive any medical treatment. One patient from each group had poor wound healing, which was characterized by persistent bleeding and exudation from the wound and was then treated with a vacuum suction device.

**Table 4:** Postoperative blood transfusion and complications

	DAA	PLA	Total
Blood transfusion	3	2	5
Complications			
Dislocation	0	2	2
Periprosthetic fracture	0	1	1
LFCN injury	3	0	3
Bursitis	0	1	1
Poor wound healing	1	1	2
Total	7	7	14

*LFCN* lateral femoral cutaneous nerve

## Discussion

Various approaches to THA have been attempted in the past decade, each with advantages and disadvantages. DAA, as a new and minimally invasive approach, has often been compared with PLA by researchers. Some studies have suggested that DAA can offer a faster recovery than PLA [24, 25]. In contrast, some studies have reported no significant difference between the two approaches in terms of postoperative rehabilitation, but have found that DAA is accompanied by a steeper learning curve and more complications than PLA [11, 26, 27]. In this study, perioperative Hb changes plus early postoperative HHS and VAS pain scores indicate that DAA results in a faster improvement of Hb after surgery, with less pain and better functional outcomes in the early postoperative stage. In contrast, DAA has a longer operative time and more blood loss than PLA.

In our study, there were some significant differences in primary outcomes between the two groups within 2 weeks post-surgery. The DAA group showed more blood loss than the PLA group during surgery. However, the lowest point of Hb in the DAA group appeared on day three after surgery, which was earlier than that of the PLA group. Subsequently, the absolute value of ΔHb of the DAA group on the fourth and fifth days after surgery was smaller than that of the PLA group, and no significant differences in ΔHb level were found between the two groups at later time points. The higher HHS and lower VAS pain score after surgery also suggested that DAA led to faster recovery. These results are consistent with previous reports which find that the DAA has certain advantages in both early postoperative recovery and rehabilitation [28, 29].

The Hb decline on the day of surgery was mainly caused by blood and fluid loss during THA. In this study, Hb decreased more in the day following surgery for the DAA group, which we suggest is associated with longer operative time. It is generally thought that DAA will cause more intraoperative blood loss, which may be reflected by our finding of a drop in Hb levels after surgery. Increased blood loss mostly occurs in the femoral side, which is related to the difficult observation of posterior capsular bleeding through DAA [28]. A study by Zhao et al. [30] which compared intraoperative blood loss for the two techniques found significant differences between DAA and PLA. In that study, the DAA group was associated with 42 mL more intraoperative blood loss as well as lower Hb levels on all the postoperative days examined. Spaans et al. [14] found that the mean blood loss in the DAA group was almost twice as much in the PLA group, while no learning effect was observed in DAA group. However, a few other reports have not supported the view that DAA results in more blood loss than other approaches. Alecci et al. [31] found no significant differences in blood loss and operation time in a cohort of 419 patients when comparing DAA with a direct lateral approach. In another study, when it was compared with PLA, DAA showed better results in terms of hospitalization, blood loss and functional scores [32].

Postoperative, recessive blood loss was the main cause of postoperative Hb decline for both groups. The mechanism of this blood loss has not yet been established. Studies have suggested that in total knee arthroplasty, around 40% of the hidden loss was due to tourniquet use leading to hemolysis during reperfusion, while 60% was due to tissue extravasation [33, 34]. In THA, while there is no ischemia-reperfusion injury as a result of surgical stress, anesthesia, or other stress reactions, free fatty acids in the body can be induced to produce a large amount of peroxide, which causes acute damage to red blood cells in the body, resulting in a hemolysis reaction [35]. For 60% of the recessive blood loss resulting from tissue extravasation, Mcmanus and colleagues [36] used the isotope labeling method in order to find a large number of labeled red blood cells in the interstitial space after surgery. This area of the red blood cells did not participate in systemic circulation, while some capillaries appeared to be abnormally open as a result of mechanical extrusion factors including prosthesis implantation and reaming.

The phenomenon of abnormal opening further increased the possibility of tissue bleeding. If joint capsules are not preserved during THA, this may lead to increased recessive blood loss [37]. In this study, the Hb decline in the DAA group was found to be slower than that of the PLA group after surgery, which suggests that there was less recessive blood loss in the DAA group. We found DAA to be associated with less recessive blood loss, which may in turn be associated with less soft tissue damage in DAA. The DAA technique utilizes the intermuscular plane and does not cut short external rotation muscles, therefore minimizing soft tissue and muscle damage. Several studies have found DAA to be associated with lower levels of creatine kinase and inflammatory markers, thus indicating that DAA causes significantly less muscle damage than PLA [30, 38]. However, no statistically significant differences of ΔHb between two groups were observed 6 weeks after surgery. Our results confirm the view of previous reports that the benefits of DAA are primarily achieved in the early stages of recovery, with long-term outcomes being similar to other approaches.

In our study, patients in the DAA group reported shorter hospital stays and better pain relief and achieved higher functional scores in the early postoperative stage, which is consistent with several previous studies [28–30]. Parvizi et al. [39] and Nakata et al. [40] compared DAA and other approaches, and found DAA to be associated with more rapid recovery of hip function and less pain during the early rehabilitation phase. A meta-analysis of DAA versus a posterior approach in THA indicated that the DAA group was associated with an increase of HHS at the 2 and 4-week time points. However, no significant HHS difference was found between the two groups at 12 weeks post-surgery. The DAA group was associated with a significant reduction of the VAS pain score at 24, 48, and 72 h [41].

DAA for surgical exposure through the intermuscular plane can theoretically spare periarticular muscles of hip and minimize muscle damage. We considered that this may be the reason why its early functional outcomes outperformed other approaches. A randomized prospective study compared muscle damage between DAA and a lateral approach using serum makers and MRI [42]. This study found that DAA resulted in less muscle damage than the lateral approach. However, muscle damage due to surgical approach had no influence on functional outcomes after three postoperative months. Meanwhile, fewer postural limitations after DAA can reduce the burden of hip joint activity in patients and improve postoperative experience. DAA also accelerated the recovery of postoperative joint function to some extent. Here, the DAA group showed a slower decrease in postoperative Hb and reached the lowest point earlier than the PLA group, which may also be related to higher early functional scores after DAA. Maezawa et al. [43] evaluated muscle strength after THA by investigating postoperative straight leg raising strength. This measure of strength was obviously higher in the patients whose 10-day postoperative/preoperative Hb ratio was > 85% at 2 months after surgery. This demonstrated that postoperative recovery of Hb may play a key role for the recovery of postoperative hip function.

Limitations of the study include the small sample size and short follow-up of both HHS and VAS pain score. Since this is a single-center study, the sample is small. A multi-center prospective study with a larger sample is needed to confirm changes of perioperative Hb in THA. A 6-month follow-up period is sufficient to observe changes in perioperative Hb levels. However, a longer follow-up is needed to determine whether there is a difference in long-term functional recovery between DAA and PLA. In addition, patients who had a significant osteoporosis or BMI > 30 kg/m^2^ were not included in this study, which is consistent with a previous study [25]. Patients living with severe osteoporosis may be at risk of fracture when using DAA for intraoperative femoral manipulation. The American Association of Hip and Knee Surgeons evidence-based committee recommends that patients with BMI > 40 kg/m^2^ do not use DAA, due to increased risk of infection, incision injury and intraoperative exposure difficulties [44]. In consideration of ethnic differences, we set inclusion criteria for this to ensure that patients’ BMI did not exceed 30 kg/m^2^. In addition, we did not use blind methods as a result of the obvious differences in the position of the surgical incision.

## Conclusions

As a result of the prolonged operative time, the decrease of Hb caused by intraoperative bleeding was greater in the DAA group. However, DAA also showed a slower decrease in postoperative Hb and reached the lowest point earlier than PLA, which suggested there was less recessive blood loss following DAA. This may be related to fewer soft tissue injuries in the DAA. Using a direct anterior approach in total hip arthroplasty results in a faster functional recovery in the early phase, with long-term outcomes being no different than those found in the posterolateral approach. By understanding changes in hemoglobin after total hip arthroplasty, we are able to better grasp the optimal timing of discharge, further decrease medical costs, reduce potential medical risks, and improve patient satisfaction. However, larger samples and multi-center randomized controlled clinical trials are needed to validate these views.

## Data Availability

The datasets during and/or analyzed during the current study are available from the corresponding author on reasonable request.

## Abbreviations

DAA: Direct anterior approach
PLA: Posterolateral approach
Hb: Hemoglobin
THA: Total hip arthroplasty
ERAS: Enhanced recovery after surgery
BMI: Body mass index
HHS: Harris hip score
VAS: Visual analog scale
SD: Standard deviation

## References

[1] Berger RA, Jacobs JJ, Meneghini RM (2004). Rapid rehabilitation and recovery with minimally invasive total hip arthroplasty. Clin Orthop Relat Res.

[2] Learmonth ID, Young C (2007). The operation of the century: Total hip replacement. Lancet.

[3] Ke-Rong D (2015). Twenty-year accelerated development of artificial joints in China. Chin J Joint Surg.

[4] Berger RA (2003). Total hip arthroplasty using the minimally invasive two-incision approach. Clin Orthop Relat Res.

[5] Kennon RE, Keggi JM, Wetmore RS (2003). Total hip arthroplasty through a minimally invasive anterior surgical approach. J Bone Joint Surg Am.

[6] SuperPath (2017). The direct superior portal-assisted total hip approach. JBJS Essent Surg Tech.

[7] Mayr E, Nogler M, Benedetti MG (2009). A prospective randomized assessment of earlier functional recovery in THA patients treated by minimally invasive direct anterior approach: A gait analysis study. Clin Biomech (Bristol, Avon).

[8] Mjaaland KE, Kivle K, Svenningsen S (2015). Comparison of markers for muscle damage, inflammation, and pain using minimally invasive direct anterior versus direct lateral approach in total hip arthroplasty: A prospective, randomized, controlled trial. J Orthop Res.

[9] Post ZD, Orozco F, Diaz-Ledezma C (2014). Direct anterior approach for total hip arthroplasty: Indications, technique, and results. J Am Acad Orthop Surg.

[10] Goebel S, Steinert AF, Schillinger J (2012). Reduced postoperative pain in total hip arthroplasty after minimal-invasive anterior approach. Int Orthop.

[11] de Steiger RN, Lorimer M (2015). What is the learning curve for the anterior approach for total hip arthroplasty?. Clin Orthop Relat Res.

[12] Lee GC (2015). Complications following direct anterior hip procedures: Costs to both patients and surgeons. J Arthroplast.

[13] Meneghini RM, Elston AS, Chen AF (2017). Direct anterior approach: Risk factor for early femoral failure of cementless total hip arthroplasty: A multicenter study. J Bone Joint Surg Am.

[14] Spaans AJ, van den Hout JA (2012). High complication rates in the early experience of minimally invasive total hip arthroplasty by the direct anterior approach. Acta Orthop.

[15] Zijlstra WP, De Hartog B, Van Steenbergen LN (2017). Effect of femoral head size and surgical approach on risk of revision for dislocation after total hip arthroplasty. Acta Orthop.

[16] Kehlet H (1997). Multimodal approach to control postoperative pathophysiology and rehabilitation. Br J Anaesth.

[17] Li J, Zhu H (2019). Enhanced recovery after surgery (ERAS) pathway for primary hip and knee arthroplasty: study protocol for a randomized controlled trial. Trials.

[18] Krebs VE, Higuera C, Barsoum WK (2006). Blood management in joint replacement surgery: What’s in and what’s out. Orthopedics.

[19] Gwam CU, Mistry JB, Etcheson JI (2018). Decline in allogeneic blood transfusion usage in total hip arthroplasty patients: National Inpatient Sample 2009 to 2013. Hip Int.

[20] Walsh M, Preston C, Bong M (2007). Relative risk factors for requirement of blood transfusion after total hip arthroplasty. J Arthroplast.

[21] Zhao H, Xiang M, Xia Y (2018). Efficacy of oral tranexamic acid on blood loss in primary total hip arthroplasty using a direct anterior approach: A prospective randomized controlled trial. Int Orthop.

[22] Kleinert K, Werner C, Mamisch-Saupe N (2012). Closed suction drainage with or without re-transfusion of filtered shed blood does not offer advantages in primary non-cemented total hip replacement using a direct anterior approach. Arch Orthop Trauma Surg.

[23] Carson JL, Terrin ML, Noveck H (2011). Liberal or restrictive transfusion in high-risk patients after hip surgery. N Engl J Med.

[24] Higgins BT, Barlow DR, Heagerty NE (2015). Anterior vs. posterior approach for total hip arthroplasty, a systematic review and meta-analysis. J Arthroplast.

[25] Putananon C, Tuchinda H, Arirachakaran A (2018). Comparison of direct anterior, lateral, posterior and posterior-2 approaches in total hip arthroplasty: Network meta-analysis. Eur J Orthop Surg Traumatol.

[26] Connolly KP (2016). Direct anterior total hip arthroplasty: Comparative outcomes and contemporary results. World J Orthop.

[27] Rykov K, Reininga IHF, Sietsma MS (2017). Posterolateral vs direct anterior approach in total hip arthroplasty (POLADA Trial): A randomized controlled trial to assess differences in serum markers. J Arthroplast.

[28] Barrett WP, Turner SE (2013). Prospective randomized study of direct anterior vs postero-lateral approach for total hip arthroplasty. J Arthroplast.

[29] Müller M, Tohtz S, Springer I (2011). Randomized controlled trial of abductor muscle damage in relation to the surgical approach for primary total hip replacement: Minimally invasive anterolateral versus modified direct lateral approach. Arch Orthop Trauma Surg.

[30] Zhao HY, Kang PD, Xia YY (2017). Comparison of early functional recovery after total hip arthroplasty using a direct anterior or posterolateral approach: A randomized controlled trial. J Arthroplast.

[31] Alecci V, Valente M, Crucil M (2011). Comparison of primary total hip replacements performed with a direct anterior approach versus the standard lateral approach: Perioperative findings. J Orthop Traumatol.

[32] Faldini C, Perna F, Mazzotti A (2017). Direct anterior approach versus posterolateral approach in total hip arthroplasty: Effects on early post-operative rehabilitation period. J Biol Regul Homeost Agents.

[33] Sehat KR, Evans R (2000). How much blood is really lost in total knee arthroplasty? Correct blood loss management should take hidden loss into account. Knee.

[34] Pattison E, Protheroe K, Pringle RM (1973). Reduction in haemoglobin after knee joint surgery. Ann Rheum Dis.

[35] Yuan T, Fan WB, Cong Y (2015). Linoleic acid induces red blood cells and hemoglobin damage via oxidative mechanism. Int J Clin Exp Pathol.

[36] Mcmanus KT, Velchik MG, Alavi A (1987). Non-invasive assessment of postoperative bleeding in TKA patients with Tc-99m RNCs. J Nucl Med.

[37] Liu X, Zhang X, Chen Y (2011). Hidden blood loss after total hip arthroplasty. J Arthroplast.

[38] Bergin PF, Doppelt JD, Kephart CJ (2011). Comparison of minimally invasive direct anterior versus posterior total hip arthroplasty based on inflammation and muscle damage markers. J Bone Joint Surg Am.

[39] Parvizi J, Restrepo C (2016). Total hip arthroplasty performed through direct anterior approach provides superior early outcome: Results of a randomized, prospective Study. Orthop Clin North Am.

[40] Nakata K, Nishikawa M, Yamamoto K (2009). A clinical comparative study of the direct anterior with mini-posterior approach: Two consecutive series. J Arthroplast.

[41] Wang Z, Hou JZ, Wu CH (2018). A systematic review and meta-analysis of direct anterior approach versus posterior approach in total hip arthroplasty. J Orthop Surg Res.

[42] De Anta-Díaz B, Serralta-Gomis J, Lizaur-Utrilla A (2016). No differences between direct anterior and lateral approach for primary total hip arthroplasty related to muscle damage or functional outcome. Int Orthop.

[43] Maezawa K, Nozawa M, Yuasa T (2018). Postoperative hemoglobin and recovery of hip muscle strength after total hip arthroplasty. J Orthop.

[44] Jahng KH, Bas MA, Rodriguez JA (2016). Risk factors for wound complications after direct anterior approach hip arthroplasty. J Arthroplast.

